# Recreation without Damnation

**Published:** 1875-07

**Authors:** Ben. H. Pratt


### THE BISTOURY.

Vol. XI. ELMIRA, N. Y., JULY, 1875. No. 2.

### Contributions.

### RECREATION WITHOUT DAMNATION.

BEN. H. PRATT.

The season waxes in warmth. The debilitating days have come, the laziest of the year. Now the merchant closes his ledger at bed time with a slam, and running his tired fingers through his thin hair, soliloquizes. He addresses himself after the manner of men, and interrogates his personality. He would know how much it profiteth a man to gain a big balance in his favor at his banker's, and loose his physical integrity. He ponders upon the gains and losses of the year past and congratulates himself upon the fact that he has not allowed his speculation to get the better of his judgment; that he has not become a bankrupt like unto his neighbor Jones, nor is obliged to "shin it" from door to door, like unto Smith, in order to meet his monthly obligations and stave off impending insolvency. These are sources of infinite satisfaction, but there is still an indefinable sense of something craved for—a release from the daily strain of business care, with its concomitant annoyances—a want of that feeling of freshness which the normal man should experience at the beginning of his business day—he is something more than tired at the close of his daily routine work. The professional man has come to a similar conclusion. The voice of the newest client has no music in it, for it portends hours of search through musty papers whose chirography shall perplex him—assures him of the necessity of reference to dry decisions upon cases of like ilk; which meditations fail to inspire him with any anticipations of pleasure such as he has experienced, in times past. He finds no spur to a pursuit which has ever had attractions for him; there is something wrong somewhere, and he concludes that he is worked out, and his system craves something more than mere rest. Another wearily drives his accustomed round and indifferently notes the pulse and tongue—mechanically asks after symptomatic knowledge and prescribes as one in a reverie, whose soul is not in his work. His toast and coffee of a morning fail to satisfy his palate—there's something wrong here—he's overworked, and the enervating days come to reveal the fact to him. Another rises from behind the sacred desk, and with a tired look and weak voice mechanically intones to dispirited listeners the hymns and prayers, while the sermon, the forced result of duty to his flock, is full of emptiness, and abounds in stiffly worded and blindly wrought sentences, which if not meaningless, are duller than silence. The teacher has left his chair, and having said adieu to his scholars, is recreating. Re-creating—that's it. He who uses his brain continually must soon wear out. We cannot so utterly defy nature as to leave recreation out of the question. The fountain cannot flow unless it be fed. Men cannot go on creating from day to day, and from week to week, and from month to month and from year to year. They cannot hope to build without materials. What man receives, that he can mould into other forms and freely give again; but when the springs fail, the streams run dry, and no amount of squeezing can produce a single drop. It is this strain upon the mentality of man in the vain endeavor to make something out of nothing, that plays such sad havoc with our generation of thinkers. Brilliancy is demanded in all departments of life. The hum-drum man is entirely overlooked in this age of sensation. It don't do, now days, for men to follow in the wake of their predecessors and do just as well as they did. Naught attracts unless it first startles. The conservatists are the mere sediment of society and business. True, they may grow as the accretions do, at the bottom, but nobody ever looks down into the depths to find them—nobody wants to stir them up and discolor the whole body politic. The veriest bubble upon the surface, gleaming in the sunlight and sparkling with beauty as it receives the pure white sunbeam, and decomposing it, gives forth the colors of the gorgeous spectrum, elicits more admiration than the unseen pearl that lies at the bottom. Men of to-day must absorb the crude, and having beautified it, must flash it before the eyes of their admiring fellows, or they are as the dregs which lie at the bottom, full of virtue, perhaps, and the real source of all the excellences we prize, but out of sight—of no account because they do not scintillate of themselves. It is this effort to dash out—this continual gushing propensity of the time—this desire to explode something that shall cause all men to look our way—it is these that are using up our vitality and playing the deuce with our constitutions.

We may berate the foibles of man and satirically point to them as the outgrowth of this and that nonsensical whim. We may waste page upon page in attempting to prove that recreation, as practiced as the present day, when we make a great fuss over our summer jaunts and sojourns, is a sham. But there is no excess of folly that has not at the bottom of it some foundation—some physically or mentally felt need. Fashion, it is true, does run our recreations into absurd follies, but in doing so she only takes up a necessity and converts it into a luxurious and injurious excess. Re-creation has become a recognized need of our nature—at least of those who have chosen such pursuits in life as require more brain work than manual labor, and this class is a large and growing one. The thinkers, in this age of telegraphs, railroads and newspapers—when the news of the world reaches us as soon as it is made—when it is disseminated widely and cheaply—when transit is easy, rapid and within the means of all—are not confined to the favored few. Hence a large majority of mankind, especially on this continent, does not earn its bread by the sweat of its brow, but rather by the wear and tear of mind. The brain, unlike the muscle, is not rested in a night. The calculators, devisers, theorizers, inventors, schemers,—those who depend upon their wits for position—are upon the rack continually. There is a fascination about their work that tempts to inordinate labor, and so absorbed do such become while pursuing, each the line of his own particular hobby, that consciousness of mental weakness does not come to them until the physical powers suddenly refuse to lend their aid to the imperious task-master, the brain, and mutiny in the body corporal suddenly assumes such proportions as to demand conciliation. Then we become aware that we have drawn upon our mind-stores to their utter exhaustion and must re-stock or go out of the business.

Nor is it mere rest that we need, when it comes to this. If it were so, we might simply determine to cease thinking, and folding our hands upon our laps, remain *in statu quo* until our equilibrium were restored, and then dive into the old routine again. But we are such slaves to association that place has more to do with thought than we will at once admit. The particular desk at which we are accustomed to do our thinking—the room in which we are wont to exercise our minds most—the very building—the town or city even—these are continual temptations to return to the pursuit of our usual avocations. Hence it is that to avoid this *in loco quo* we go off to re-create. The muscle worker *rests* at home—the brain worker cannot *re-create* except he fly his associations and throw his mind off the old track. We can command our muscle to be idle and it will obey—we may issue a thousand anathema maranathas against our brain to warn it not to think, but there will be no obedience unlese we remove it whither it will be distraught by other and lighter subjects of contemplation. This is why the brain worker goes abroad; this is why, when the enervating season approaches, he is warned of the necessity of re-creation, and seeks out some location as nearly the antipodes of his accustomed haunts as possable. Hence the Sea Side, the Springs and the Mountains are the resorts of such—hence the hegira from our cities in July.

How tolerable this would be, had not Society and Fashion converted this necessity of ours into a by-word, a reproach. How sensible a relief to overworked brains is utterly shorn of all its redeeming qualities by those who, not having brains, and hence needing no recreation, imagine they, too, must trundle off somewhere during the summer months. And having no earthly object in view save that of being fashionable, they must trump up something in the way of an excuse, and invent imaginary ills, for which they become self-prescribers, and their prescriptions run somewhat after this fashion:

℞

Trunki magni Saratogaensis, DC lbs.
Togarum moire-antiquarum, VIII.
Togarum silkarum, XIV.
Garmentarum albarum, quant. suf.
Capitis ornamenti, innumerabili.
Adde fixems ad libitum.
Omnia, sed primum, septies diem, applicata esse et cum vino et spiritu frumenti et cibo delicioso, sine compunctione capta esse.

Alas! to the shame of otherwise sensible men, and women, be it said, they are slaves to that empty headed goddess Society, and go whithersoever she trapses—dally where she dallies—giggle and primp and adorn themselves at her nod and dictation—suffer themselves to be led *per naso* to their own disgust.

Why don't somebody, with a soul above dress and tawdry gentility, and silly watering place platitudes, build a big hotel somewhere, on the common sense plan, advertise the fact that the patrons thereof will not be compelled to dress more than once a day—that the he snob and the she snob will not be tolerated—that roast beef and mutton chops can be partaken of in business suits and calico—that no dancer will be ostracised because he prefers to do his jig in his shirt sleeves—that dimity aprons and linen collars and cuffs, if clean, will constitute the chief ornament of the drawing room costume—that low necks will be construed as a vulgarity and kid gloves be reckoned a bore—that freckles and tan shall be honorable marks of the wearer's contempt for the conventional delicacy ascribed to woman—that fuss shall be ignored, and fun, frolic and fatigue shall sauce the plainer meats with the appetite that waits upon healthful digestion—that corsets and tight shoes shall be left at home along with the other penitential tortures which Society requires folks to periodically inflict upon themselves. Why, we ask, won't some philanthropic Crœsus institute such a summer resort, and bankrupt some of the debilitating concerns now recognized as the sources whence come, dyspepsias, anæmias, consumptions, bankruptcies, run-away matches, abductions, seductions, ruinations of character, gambling habits, delirium tremens, *et id omne genus?* Let us have recreation without damnation, physical and moral. Let us, until some such place is provided, take ourselves and families to some quiet spot—whether in the highlands, or along the ocean shore, or by the side of some healing waters, as our tastes suggest—and there re-create as nature intends men shall recreate, by a relief from wonted care and brain-work, and thence, thoroughly invigorated by a communion with nature, return home refreshed in body and mind, while we are unmarred as to our morals and unprejudiced as to our estate.

